# Innovative drugs, chemicals, and enzymes within the animal production chain

**DOI:** 10.1186/s13567-018-0559-1

**Published:** 2018-07-31

**Authors:** Yousef I. Hassan, Ludovic Lahaye, Max M. Gong, Jian Peng, Joshua Gong, Song Liu, Cyril G. Gay, Chengbo Yang

**Affiliations:** 1 Guelph Research and Development Centre, Agriculture and Agri-Food Canada, Guelph, ON Canada; 2 Jefo Nutrition Inc., Saint-Hyacinthe, QC Canada; 3 Department of Biomedical Engineering, Wisconsin Institutes for Medical Research, University of Wisconsin-Madison, 1111 Highland Avenue, Madison, WI 53705 USA; 4 College of Animal Science, Huazhong Agricultural University, Wuhan, China; 5 Department of Biosystems Engineering, University of Manitoba, Winnipeg, MB Canada; 6 Office of National Programs, Animal Production and Protection, Agricultural Research Service, US Department of Agriculture, Beltsville, MD 20705 USA; 7 Department of Animal Science, University of Manitoba, Winnipeg, MB Canada

## Abstract

The alarming number of recently reported human illnesses with bacterial infections resistant to multiple antibacterial agents has become a serious concern in recent years. This phenomenon is a core challenge for both the medical and animal health communities, since the use of antibiotics has formed the cornerstone of modern medicine for treating bacterial infections. The empirical benefits of using antibiotics to address animal health issues in animal agriculture (using therapeutic doses) and increasing the overall productivity of animals (using sub-therapeutic doses) are well established. The use of antibiotics to enhance profitability margins in the animal production industry is still practiced worldwide. Although many technical and economic reasons gave rise to these practices, the continued emergence of antimicrobial resistant bacteria is furthering the need to reduce the use of medically important antibiotics. This will require improving on-farm management and biosecurity practices, and the development of effective antibiotic alternatives that will reduce the dependence on antibiotics within the animal industry in the foreseeable future. A number of approaches are being closely scrutinized and optimized to achieve this goal, including the development of promising antibiotic alternatives to control bacterial virulence through quorum-sensing disruption, the use of synthetic polymers and nanoparticles, the exploitation of recombinant enzymes/proteins (such as glucose oxidases, alkaline phosphatases and proteases), and the use of phytochemicals. This review explores the most recent approaches within this context and provides a summary of practical mitigation strategies for the extensive use of antibiotics within the animal production chain in addition to several future challenges that need to be addressed.

## Introduction

The rising number of recently reported human illnesses with bacterial infections resistant to multiple antibacterial agents has become a fundamental concern in recent years [1, 2]. This phenomenon has become a serious concern with regard to the emergence of antibiotic-resistant bacteria called “superbugs” [3, 4], especially when considering that humans only started using antibiotics in the past century with Sir Alexander Fleming’s discovery of penicillin in 1928. Since then, the extensive use of antibiotics has formed the cornerstone of modern medicine. The benefits of antibiotics (as medications) attracted the same attention in the field of animal treatment/veterinary medicine [5], but since the early 1960s and 70s they have grown in appeal as growth promotion agents (when used at sub-therapeutic doses) for enhancing animal productivity [6]. In such a practice, antibiotics intended for medical use are used as growth promotors through their incorporation in feed at low doses to promote animal productivity, rather than using them for the intended purpose of treating a bacterial infection. While this practice was completely banned in certain countries (banned in Europe since 2006 according to the 1831/2003/EC legislation), it is still in practice in large parts of the world.

The use of antibiotics for growth promotion has resulted in a significant increase in the reported number of antibiotic-resistant bacteria found within the animal production chain. For instance, a recent study reported that a high percentage of *Campylobacter* spp. found within the beef food system in Malaysia were resistant to tetracycline (76.9%) and ampicillin (69.2%), respectively [7]. Similarly, the inspection of chicken-meat samples obtained from Bharatpur, Nepal; revealed the prevalence of multidrug resistant isolates with levels close to 80% of the tested samples [8]. Likewise, the spread of *Salmonella* within the poultry industry in China was probed most recently and a total of 170 non-duplicate isolates were recovered. The retrieved isolates showed resistance to several antibacterial agents including ciprofloxacin (68.2%), amikacin (48.2%) and cefotaxime (44.7%) [9].

The widespread practice among farmers and producers of adding antibiotics to feed to enhance their profitability margins and decrease their losses due to challenges faced within the production chain, is unfortunately here to stay [10]. Rather than forcing changes to the current practice (which would be an uphill battle), identifying antibiotic alternatives that can alleviate the issues surrounding antibiotics use is a strategy that might address this chronic dilemma [11]. This review explores the most recently published literature in regard to antibiotic alternatives and provides a summary of possible mitigation strategies within the animal production field.

## Anti-bacterial virulence drugs

One of the most promising approaches for controlling bacterial pathogenicity is to target their virulence mechanisms. In this approach, the developed drug disarms the pathogen and eliminates its ability to infect host cells, rather than killing the bacterium or stopping its growth [12]. Plausible targets for bacterial virulence disruption are: bacterial two-component systems, bacterial biofilm formation mechanisms, bacterial capsulation systems, bacterial toxins secretion systems, protein secretion mechanisms, cyclic di-GMP signaling mechanisms, and quorum-sensing mechanisms, with the last being the favored empirical target.

### Quorum-sensing disruption

Population density is a major factor that influences bacterial growth and response. In general, a stimulus from the surrounding environment usually leads to a highly-coordinated response among all the bacterial cells within the entire population that restricts the expression of certain genes/clusters of the genome, leading the entire population to an optimum outcome (survival) of all involved cells [13].

As mentioned earlier, microbes use quorum-sensing to orchestrate collective population behavior including biofilm formation and/or virulence factors secretion. Since the entire process of quorum-sensing is dependent upon the production and release of specific chemicals/signals at a population-wide scale, it is theoretically plausible to disrupt this entire process by interfering with the production/accumulation of such signaling molecules (known also as auto-inducers). In principle, chemicals involved in quorum-sensing disruption can overcome antibiotic resistance by disarming pathogens of virulence factors that facilitate disease/infection progression while leaving bacterial growth pathways untouched. Either as stand-alone medications or administrated in parallel with antibiotics, these drugs are intended to treat bacterial infections in a largely pathogen-specific manner [14].

The use of natural products (or their derivatives) to target quorum-sensing mechanism(s) of pathogenic bacteria was among the most frequently tested antibiotic alternatives in the past two decades within the context of controlling the virulence of animal/human pathogens [15]. In a recent study, phytochemicals derived from multiple medicinal plants of the *Myrtaceae* family were investigated for their ability to influence the production of a specific virulence factor in *Pseudomonas aeruginosa*. Both *Syzygium jambos* and *S. antisepticum* demonstrated the ability to inhibit the production of pyoverdine, a well-established virulence factor, in *P. aeruginosa* ATCC 27853 [16]. A subsequent detailed analysis revealed the presence of three phytochemicals (phytol, ethyl-linoleate and methyl-linolenate) that were able to significantly lower pyoverdine production and influence the proteolytic and haemolytic activities of *P. aeruginosa* [16].

**Figure 1:**
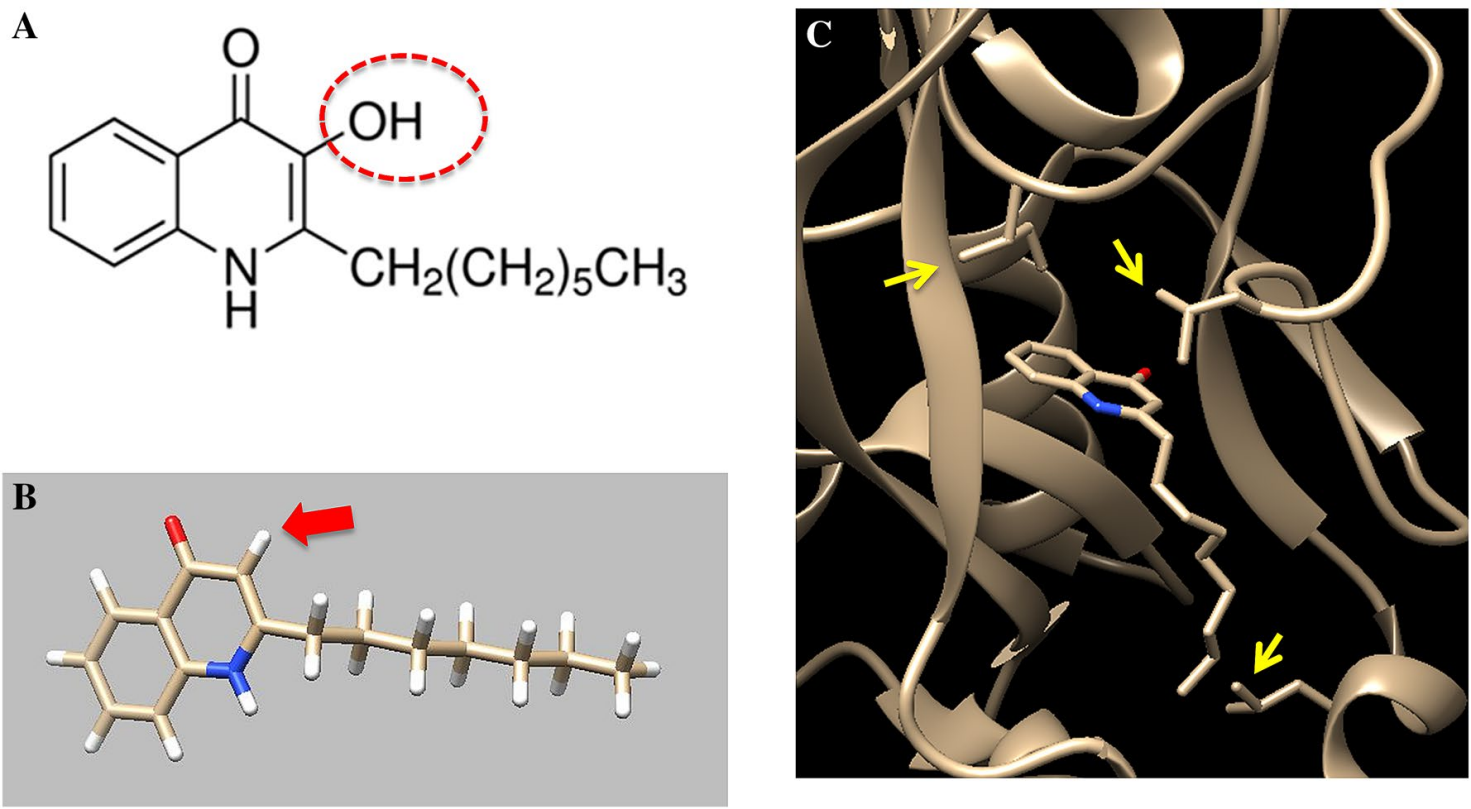
**PqsR transcriptional regulator interactions with substrate(s) and inhibitor(s). A** The chemical formula of a natural binder of the PqsR transcriptional regulator, 2-heptyl-4-hydroxyquinoline, **B** the hydroxyl group at the position-3 of 2-heptyl-4-quinolone (HHQ) has a pivotal role in PqsR and HHQ interactions, **C** the different amino acids side-chains involved in establishing the PqsR transcriptional regulator interactions and controlling the production of pyocyanin in *Pseudomonas aeruginosa* at a subsequent stage.

Within the same context, a number of synthetic compounds involved in modulating *P. aeruginosa* quorum-sensing regulator (PqsR) activity, a transcriptional regulator that controls the production of many virulence factors (including pyocyanin), were inspected [17]. All tested compounds shared a similar structure to the natural ligand 2-heptyl-4-quinolone (Figure 1A) and showed four different activity-profiles (namely agonists, antagonists, inverse agonists and biphasic). The most influential factor was the substituent(s) in position 3 of the quinolone core (Figure 1B), which acted as a switch between the different profiles. In depth molecular-docking and examination revealed that each of the PqsR modulators showed distinctive molecular interactions within the PqsR binding domain (Figure 1C). Furthermore, the shift between profiles was attributed to the ability of such substituents to either accept or donate a hydrogen bond or to form hydrophobic interactions. Ultimately, the strongest inhibition of pyocyanin production was associated with the inverse agonists profile [17].

More recently, an autoinducer 2 (AI-2) that is produced in several pathogenic *Vibrio* species (including *Vibrio harveyi*, *V. campbellii*, and *V. parahaemolyticus*) and shown to regulate the virulence of the *Vibrio* species towards *Artemia franciscana* (brine shrimp), was targeted with one natural [(5*Z*)-4-bromo-5-(bromomethylene)-3-butyl-2(5*H*)] and one synthetic [(5*Z*)-4-bromo-5-(bromomethylene)-2(5*H*)] furanones [18]. The inclusion of both compounds in low concentrations protected *Artemia* from the *V. harveyi* strain BB120 and *V. campbellii* strain LMG21363 in a concentration-dependent manner.

Moreover, *V. harveyi*, the pathogen responsible for vibriosis outbreaks in *Macrobrachium rosenbergii* (giant freshwater prawn), was targeted recently with three quorum-sensing disruptors including cinnamaldehyde, (Z-)-4-bromo-5-(bromomethylene)-2(5*H*)-furanone and (Z)-4-((5-(bromomethylene)-2-oxo-2,5-dihydrothiophen-3-yl)metoxy)-4-oxobutanoic acid [19]. None of the tested disruptors affected *M. rosenbergii* larvae growth yet they were all able to positively increase the larvae survival rates in response to *V. harveyi* challenge when added to the culture water (at 1 μM final concentration).

Other cellular targets such as the SaeRS two-component systems in *Staphylococcus aureus* were suggested as good targets for possible virulence control [20, 21], as the case of norlichexanthone, a small non-reduced tricyclic polyketide which is mainly secreted by fungi and lichens. Norlichexanthone was used for reducing the expression of the *hla* gene encoding α-hemolysin as well as the regulatory RNAIII of the *agr* quorum-sensing system in *S. aureus* 8325-4 and USA300 strains. Upon close inspection, norlichexanthone was found to reduce *S. aureus* toxicity towards human neutrophils; to interfere with AgrA, an *agr* two component response regulator, binding to its DNA targets; and to reduce staphylococcal biofilm formation [22]. A further transcriptomic analysis revealed that the genes regulated by the SaeRS two-component system were in fact repressed by norlichexanthone in comparison to the untreated cells [22].

While the scientific community has developed a good grasp of the mechanisms involved in bacterial quorum-sensing and revealed many groups and classes of either naturally-available or synthetic compounds that can be utilized in pioneering this approach of controlling bacterial pathogenesis, multiple challenges still exist for its wide-spread implementation [23–25]. Beyond the initial costs of optimizing the above compounds/chemicals in regard to solubility, tissue/organ accessibility, target-delivery, and efficacy issues; the existence of other natural chemicals that can attenuate the used signals/chemicals is a possibility. Moreover, the ability to specifically target one pathogen without disrupting the natural rhythm of indigenous microbiota, given the large number of bacterial species that employ similar quorum-sensing communication systems, needs to be considered. This is a true concern as many of the chemicals used in quorum-sensing disruption have some off-target capabilities and can possibly attenuate the activities of some desirable bacterial species, e.g. lactic acid bacteria, that otherwise would exert a protective effect in the long run.

Furthermore, the initial promise of disrupting mechanisms involved in bacterial quorums is based on the assumption that such anti-virulence drugs will minimize any resistance-development issues within the targeted bacterial species; such claims were considered unfounded most recently through the emergence of reports showing that resistance towards anti-virulence compounds does exist [12]. Delineating the involved mechanisms and probing the relationships between structure–functionality might be the way for addressing any long-term resistance issues with quorum-sensing disruptors.

## Chemicals

### Organic acids and their derivatives

For quite some time, organic acids have been known for their ability to prevent food spoilage and extend the shelf-life of many perishable commodities [25]. This capability brought attention to their possible usage in combating bacterial diseases and fighting infections. Specific organic acids such as benzoic and butyric acids (and derivatives) have extensively been used in controlling bacterial contaminations and eradicating pathogens. The potency of these acids is dependent on the physiological status of the targeted microorganisms and the physicochemical characteristics of the surrounding environment [26].

A recent study inspected the effects of three commercial formic and propionic acid preparations on the growth performance, cecal microbiology, and immunity of broiler chickens challenged with *Escherichia coli* F4 (K88)^+^. The retrieved results suggested that dietary organic acids can restore the growth and immunity parameters of *E. coli*-challenged broilers to the original levels noticed in non-challenged birds [27].

Another experiment was conducted using Ross 308 broiler chicks to study the effect of using two commercial mixtures of organic acids as a substitute for a commercially available antibiotic growth promoter. Birds that were fed the organic acids-supplemented diets had gains of 3–16% in comparison to the negative control group (which had no organic acids or antibiotic supplements) with improved feed conversion ratios [28]. Furthermore, the used organic acids increased the dressing percentage and bursa weight among treatment birds without affecting the liver, spleen and thymus-weights. Interestingly, all of the organic acid preparations decreased intestinal *E. coli* and *Salmonella* while the used dietary antibiotic, enramycin in this case, was efficient in decreasing *Salmonella* counts only. Differences, however, were noticed among individual organic acid mixtures that were tested for either their influence on birds’ performance or intestinal bacterial counts [28].

How short-chain fatty acids might influence the ability of *Salmonella* Enteritidis to invade avian intestinal epithelial cells was investigated at an earlier time [29]. The pre-incubation of *Salmonella* Enteritidis with various concentrations of propionate or butyrate resulted in decreased invasions compared to the control group. Later, butyrate was found to specifically down-regulate *Salmonella* pathogenicity island 1 (SPI1) genes expression including hilD and invF [30].

Based on the research outlined above, it was suggested that diets that stimulate organic acid production (short and medium-chain fatty acids) within the caecum can possibly help control *Salmonella enterica* infections in a very cost-effective way. Alternatively, the addition of acids to the feed or drinking-water could be another effective approach to control these infections [31].

Recent animal trials conducted on young chickens challenged with *Salmonella* Enteritidis involved feeding them with short-chain fatty acids encapsulated in mineral carriers, resulting in a slow release during the transport of these carriers throughout the intestinal tract. This treatment was able to significantly decrease the colonization and the invasion capacity of this pathogen [32].

The protection of the organic acids through the use of lipid matrixes, polymers, and/or enteric coating was shown to prevent their dissolution or disintegration within the gastric environment and to maximize their efficacy and bio-potency. An experiment conducted to investigate the effects of protected essential oils and organic acids in poultry (as antibiotics replacement) produced encouraging outcomes showing that the protected essential oil and organic acid mixtures could indeed serve as efficient growth-promoters and antibiotic alternatives [33]. Surprisingly, a subsequent bacterial species analysis of the intestinal tract revealed that the protected organic acids led to some fundamental changes within the gut microbiota, mainly in regard to the number of observed *Lactobacillus* species [33]. This suggests that the used organic acids carried some indirect beneficial effects in addition to the direct ones.

Blends of protected organic acids were used in a 2-week trial to evaluate their effects on growth performance, nutrients digestibility, intestinal microbiota, and fecal gas emission. The dietary inclusion of protected organic acid blends (at 0.1–0.2% levels) linearly improved average daily gains and nutrients digestibility while decreasing fecal ammonia and acetic acid emissions. Furthermore, the supplementation with protected organic acids affected meat quality leading to a significant increase in the *longissimus* muscle area. Interestingly, these acids were associated with positive changes in the color and firmness of the resulting meat. Finally, the protected organic acid blends caused a reduction in the total fecal *E. coli* counts while increasing *Lactobacillus* counts in a fashion that was dependent on the dosage of the organic acid blend. The 0.2% level of protected organic acids showed more positive effects on the above production parameters and was found to be superior to the lower levels of inclusion (0.1%) [34].

One emerging concern related to the use of organic acids as antibiotic alternatives is their ability to enhance the survivability of acid-sensitive pathogens exposed to low pH by the induction of an acid tolerance response [25], which may also be linked to increased virulence. For this reason, it will be important to closely examine the behavior of pathogens under the actual production environment using the readily available molecular tools to specifically understand the genetic mechanisms that regulate bacterial response towards organic acids before developing any commercial/empirical applications at the farm gates [25, 35].

The possibility of using short chain fatty acids as alternative growth promoters is indirectly supported by the results of numerous research studies aimed at understanding the course of antibiotic treatments in animals. For example, the effect of streptomycin on intestinal microbial communities was recently tracked [36]. The results indicated a depletion of butyrate-producing clostridia from the intestinal lumen during the course of antibiotics administration. This in turn led to a noticeable decrease in total butyrate levels and a substantial increase in epithelial oxygenation later connected with the aerobic expansion of *Salmonella* Typhimurium [36]. The restriction in *Salmonella* growth/expansion was only restored after implementing a treatment with tributyrin, an ester composed of butyric acid and glycerol. The above study suggests that increasing intestinal short- and medium-chain fatty acid levels (e.g. butyrate) might indirectly limit pathogen expansion by limiting epithelial oxygenation and the aerobic growth of pathogens.

Based on the above hypothesis, a dietary intervention was optimized and recently investigated to inspect the effect of administrating protected calcium butyrate on nutrient digestibility and growth parameters in broiler chickens [37]. The inclusion of protected calcium butyrate (0.2–0.4 g/kg of finished feed) improved feed conversion ratios, body weight gains, and fat digestibility. Surprisingly, birds from the treatment group were characterized by having thick intestinal mucosa that explained the observed improvements in digestion and absorption of feed nutrients hence the birds’ overall enhanced performance [37].

A similar study was conducted to investigate the effects of benzoic acid on the intestinal development and growth parameters of farm animals. A basal diet was supplemented with either 2000 or 5000 mg/kg of benzoic acid [38]. Improvements in final body weights, daily growth and feed conversion ratios for these animals in addition to parallel decreases in the activity of plasma diamine oxidases and pH values of jejunal contents were attributed to the benzoic acid supplementation. Further positive changes within the intestinal microbiota were noticed through the process with increases of ileal *Bacillus* populations, decreases in *E. coli* counts, and higher *Lactobacillus* counts within the ileum and caecum of treated animals. Similar outcomes with regard to the intestinal microbial communities’ composition and the influence of medium-chain fatty acids (caprylate more specifically) on *Salmonella* Typhimurium and coliforms were reported by Messens et al. [39]. At the cellular level, the benzoic acid supplementation was also reported to increase the ratio of villus height to crypt depth and influence cellular growth-stimulating factors (such as insulin-like growth factor-1, insulin-like growth factor-1 receptor, and tight junction proteins including zonula occludens-1) mRNA levels positively though subsequent up-regulations [38].

The impact of medium-chain fatty acids and short-chain organic acids on animal gut morphology and its immune system was specifically established through a study that investigated the effect of using 0.41% fumaric and 0.32% lactic acids (short-chain organic acids) alone or in combination with 0.15% caprylic and capric acids (medium-chain fatty acids). Tissue samples were used to investigate the potential impact of these additives on villus length and crypt depth of the jejunum and to quantify intra-epithelial lymphocytes. While this study did not report any significant changes in the morphometric data, it noticed that the short-chain organic acids significantly increased the quantity of CD2^−^ CD8^−^ γδ T cells within the jejunum epithelium and confirmed that the majority of the intra-epithelial lymphocytes were expressing the surface marker CD3. Hence, the study concluded that short-chain organic acids were indeed exerting a beneficial effect on the local immunity of animals by merely evoking mechanisms known to be involved in defeating infections [40]. The caproic, caprylic, and capric acids were evaluated in a second study for their abilities to control *Salmonella* Enteritidis in chickens. Low concentrations of these acids inhibited bacterial growth and invasion in in vitro models, with caproic acid showing the highest potency. The conducted mechanistic studies indicated that these acids suppressed the expression of *hilA*, a key regulator related to the invasive capacity of *Salmonella*. The addition of caproic acid to chicken starter feed in a subsequent animal trial (in the amount of 3 g/kg) indicated a substantial decrease in *Salmonella* Enteritidis colonization levels inside the ceca and other internal organs. This result provided direct evidence of the potential use of such acids in reducing *Salmonella* colonization and the number of contaminated eggs entering the food-supply chain [41].

The minimal inhibitory concentrations (MIC) of formic, acetic, propionic, butyric, caproic and caprylic acids against 54 porcine *Salmonella* Typhimurium field-strains were recently determined [42]. These values were found to be pH-dependent as the MIC values of the tested fatty acids increased in increasing pH-values of the medium. This was especially evident in the case of formic acid which showed a large range of MIC values at pH = 4 and 6. The propionic acid was less influenced by changes in the medium’s pH suggesting that the nature of the used fatty acid (short chain fatty acids versus medium-chain ones) plays a pivotal role in the witnessed inhibitory activity [42]. The expression levels of many virulence factors such as *fimA* and *hilA* (hence strain invasivity) were significantly lowered in the presence of these acids, especially in the case of caproic or caprylic acids (2 mM as final concentrations). Furthermore, serving the animals feed supplemented with coated butyric acid decreased the levels of fecal shedding and intestinal colonization of *Salmonella* Typhimurium. Surprisingly, the uncoated fatty acids did not influence the noted fecal shedding or intestinal colonization [42].

Besides the ability of medium-chain triglycerides and fatty acids to provide instant energy and physiological benefits, they were also found to affect and stabilize the composition of intestinal microbiota and inhibit coliforms and *Salmonella* growth. The above benefits were confirmed in piglets recently by adding up to 15% medium-chain triglycerides to feed mixtures without detecting any negative impact on feed intake or any sensory/organoleptic issues associated with the finished feed. These diets resulted in lower mortality rates and better development of newborn animals, particularly underweight ones [43].

### Minerals

Poor development and growth, diseases, morbidity and mortality of animals are often caused by poor gut health associated with a leaky gut, intestinal atrophy, infections and inflammation, particularly when the animals are young [44]. In response to this problem, different remedies (including the administration of antibiotics as growth-promoters and the inclusion of organic minerals) have been used and are highly recommended for restoring natural balances to distressed animals [45, 46]. Many previously published studies reported on the feasibility of using minerals such as zinc oxide and copper salts (in elevated doses) for combating stress symptoms, boosting daily weight gain, and decreasing feed conversion ratios [47].

Zeolites, porous-, and cation-rich alumino-silicates were suggested as poultry feed additives with multiple productivity benefits. Most recently, diets of laying hens were supplemented with zeolites at 1, 2, or 4% (w/w) for 23 weeks. Cloacal swabs followed by next-generation sequencing of 16S rRNA genes reflected a significant reduction in *Enterobacteriaceae* demonstrating the ability of zeolites to decrease poultry pathogen loads without disturbing the natural beneficial gut microbiota of the animals [48]. Similarly, the effect of supplementing feed with clay minerals on the circulation of CMY-2 β-lactamase gene in commercial farms/animals was investigated and the obtained results indicated that clay supplementation may reduce the clonal diversity of blaCMY-2-positive bacterial isolates [49]. Such a practice may limit the spread of antimicrobial resistance between and within batches of animals.

Biotite and bentonite, two phyllosilicate minerals with industrial applications, were reported with several beneficial activities as antimicrobial agents. The bacterial clearance capabilities of a mixture of biotite and bentonite were investigated on experimental infection models of *Salmonella* Typhimurium through administering 1% or 2% (w/w) biotite and bentonite mixtures in feed [50]. The outcomes indicated that the clinical signs associated with a *Salmonella* Typhimurium infection and the numbers of viable bacterial cells retrieved from feces/tissues samples were significantly decreased within the treatment groups, with the 2% (w/w) supplementation level being the most promising one.

The effectiveness of replacing antibiotics (serving as growth promoters) with tribasic copper chloride and copper sulphate pentahydrate in duck diets was recently investigated [51]. After a 6-weeks feeding trial; the average daily feed intake, body weight, average daily gains, and mortality of birds were not affected by the supplementation. All production parameters were comparable to the control group which was served a basal-diet containing antibiotics (40 mg zinc bacitracin/kg and 40 mg garlicin/kg). Increases in the copper content of liver tissues in addition to iron and zinc depositions within breast muscles were noticed. Some changes in the serum activities of superoxide dismutase, glutathione peroxidase, malondialdehyde levels, serum low-density lipoprotein, and cholesterol concentrations were also observed within the treatment animals [51].

Among the many studied metal antimicrobials (such as silver, titanium, and copper oxides), only zinc oxide (ZnO) has been successfully used in animal feed at a large-scale to boost growth. The use of ZnO in these applications with much higher doses (as a therapeutic agent to replace antibiotics for weaned animals) surpasses its role as a micronutrient for optimal animal growth. The used pharmacological doses of ZnO (within the 2000–3000 mg/kg range) have proven to be effective in reducing the symptoms of post-weaning diarrhea in pigs [52, 53]. The impact of high dietary zinc oxide on the development of the intestinal microbiota was recently explored in depth in weaning piglets [54]. The presented data showed that dietary zinc at high concentrations (2425 mg/kg) leads to transient and lasting effects on the intestinal microbiota of treatment animals. The metabolic activity of the involved bacterial species was also modulated with a noticeable reduction of bacterial-metabolites concentrations and increased molar acetate ratios at the expense of propionate in the proximal intestine [54]. These changes were also associated with pronounced reductions in the *Enterobacteriaceae* and the *Escherichia* groups while *Bifidobacterium*, *Enterococcus*, *Streptococcus*, *Weissella* spp. and *Leuconostoc* spp. did not reflect any influence due to high-dietary zinc concentrations throughout the 5-week trial period.

Zinc oxide is currently used extensively in Europe as an alternative for colistin in weaned piglets and not just as a feed ingredient to improve feed conversion, as previously mentioned. In fact, new formulations of zinc oxide nanoparticles are being investigated as substitutes for the commonly used colistin sulfate. Most recently, a study indicated that the supplementation of 1200 mg/kg nano-ZnOs (approximately 30 nm in diameter) significantly increased the final body weight and average daily gain of treated animals in comparison to the colistin sulfate group (20 mg/kg). This treatment also improved the intestinal morphology, significantly decreased total aerobic bacterial populations in mesenteric lymph nodes, and significantly decreased plasma and tissue zinc concentrations (liver and tibia) [55].

Despite the promising outcomes of using high concentrations of mineral within the animal production chain, some major criticisms directed towards the use of such metals exist. The fact that many of the used heavy metals tend to accumulate in the soil resulting in serious environmental and health consequences is at the top of this list [56, 57]. Furthermore, many research teams consider such metals to be part of the antibiotic resistance problem itself as they indirectly impact the development of the antimicrobial resistance through their regulation of bacterial gene-expressions [47, 58]. There is also the risk of co-resistance selection through the use of heavy metals as most of the metal resistance genes co-localize to the same genetic elements as certain antimicrobial resistance genes [59, 60]. Recent studies indicate that this has likely resulted in the appearance and the worldwide-spread of methicillin-resistant *S. aureus* (MRSA) associated with farmed animals including pigs and poultry [61]. Other adverse effects of the use of zinc oxide have also been reported such as its negative interactions with other feed ingredients [57] causing post-weaning anemia [62] and mutagenicity-induction in mammalian cells [63]. The emergence of new formulations/conjugation methods (to nanoparticles for example) as well as micro-encapsulation technologies (e.g. fat matrix that endure higher bio-availability hence much lower final concentrations can be used), might collectively aid in addressing some of the problems connected with utilizing metals and organic-minerals within the animal production field.

### Phytochemicals

Phytochemicals are natural compounds/secondary-metabolites derived from botanicals and medicinal plants and generally used in complementary or alternative medicine to improve human health and/or treat human diseases. Certain phytogenic compounds (and their blends) have been reported to have promising anti-microbial properties [64] because of their abilities to increase the permeability of bacterial cell membranes and/or kill certain bacterial species [65]. Therefore, few selected phytochemicals attracted the attention of the animal industry as potential alternatives to antibiotics/growth-promoters.

Recently published in vitro studies have shown that thymol, carvacrol, cinnamaldehyde (or combinations) have the ability to inhibit *E. faecalis* and *E. coli* growth [66]. Furthermore, the effect of these phytochemicals on intestinal microbiota and animal performance (mainly broilers and pigs) has been evaluated in several in vivo studies. The obtained results clearly support the notion that these compounds can have a positive influence on intestinal microbiota. For instance, serving a diet supplemented with a 100 mg/kg carvacrol:thymol (1:1) blend significantly reduced the counts of bacteria belonging to the *Escherichia* and *Enterococcus* genera and improved the total counts of *Lactobacillus* within the jejunal digesta of weaning animals [67]. In another study, the cecal populations of *E. coli* showed a substantial decrease in numbers when animals were fed a diet supplemented with 100 or 150 mg/kg essential oils containing 14.5% thymol and 3.5% cinnamaldehyde, respectively, while the proportion of *Lactobacillus* species was increased [68]. Similarly, it was shown that a dietary supplementation of phytochemicals composed of 15 g/tonne thymol and 5 g/tonne cinnamaldehyde led to a significant increase of the cecal proportion of both *Lactobacillus* and *E. coli* species in broilers after 41 days of feeding [69].

Allicin is another potent phytochemical with potential applications within the animal production field. Its possible use in aquaculture against fish pathogens was recently investigated. A 10% (v/v) solution of allicin in dimethyl sulfoxide showed MICs of 125 μg/mL against *Aeromonas hydrophila* and 63 μg/mL against *Streptococcus iniae*. The utilized preparations showed a high toxicological effect on tilapia (*Oreochromis niloticus*) fingerlings while adult tilapia could tolerate the aforementioned toxic effect [70]. Further observations and investigations are needed before reaching any conclusions.

The effect of dietary allicin on the health and growth performance of weanling piglets was explored by comparing a control group that was fed a diet supplemented with antibiotics with multiple treatment groups that were fed diets supplemented with allicin (25% v/v pure allicin oil) at concentrations of 0.10, 0.15, 0.20 and 0.25 g/kg, respectively. The results showed that the average daily weight gain increased as the level of dietary allicin increased while the incidents of diarrhoea in treatment piglets decreased. Interestingly, an improvement in the local farm environment was also reported as the feces produced by the animals receiving the allicin treatment was less attractive to flies [71].

Mechanistically speaking, allicin was reported to protect intestinal cells (such as IPEC-1) from membrane damage and from the increased membrane permeability associated with infections caused by enterotoxigenic *E. coli* F4 (K88)^+^. Allicin protection was not due to its antibacterial activity, since enterotoxigenic *Escherichia coli* growth was unaffected by the presence of allicin [72].

The efficacy of garlic and allicin in treating experimental shigellosis in rabbits has also been tested. Aqueous extracts of garlic (*Allium sativum*) and allicin both showed significant in vitro and in vivo antibacterial activities against isolates of multiple drug-resistant *Shigella dysenteriae* 1, *S. flexneri* Y, *S. sonnei* and enterotoxigenic *E. coli*. Allicin aided in fully curing the infected rabbits within 3 days eliminating the challenging bacteria, judged by rectal swabs within the 2^nd^ day of treatment. Moreover, allicin did not show any adverse effects on standard blood parameters/animal performance when used at the reported therapeutic doses [73].

The ability of allicin to reduce *Campylobacter jejuni* colonization and shedding in broilers was also investigated. While the in vitro testing showed that allicin was capable of reducing *C. jejuni* numbers below the detection level at 7.5 mg/kg, the positive effect was not reflected in the treatment animals that were administrated allicin through the drinking water. Arguably, the activity of allicin was assumed to be thwarted by the presence of mucin-containing mucus [74].

While many studies highlighted the beneficial effects of phytochemical supplementation (mainly thymol and cinnamaldehyde) on animal productivity especially in broiler chickens [69], a few other studies found that the reported positive effects connected with feeding phytochemicals should be scrutinized more closely as the experimental animals reflected some conflicting/varying outcomes [75]. For example, while a diet supplemented with a 100 mg/kg carvacrol:thymol (1:1) blend exerted a positive effect on the animals’ intestinal microbiota, it did not improve the overall performance of the growing animals [67]. In another study, the performance of weaning pigs was not improved by providing a diet supplemented with 5% (wt/wt) carvacrol, 3% cinnamaldehyde, and 2% capsicum oleoresin at 300 mg/kg levels [76, 77] or a diet containing 1000 mg/kg phytochemical blends consisting of cymene, terpinene, and carvacrol (as 60% active fractions) [78]. Other studies reported an increase of the screened *Escherichia* species due to the supplementation of phytochemicals [69].

The variation of animal response toward dietary phytochemicals might be explained by the dependence of outcomes on many factors including treatment duration, the administered dose, and how these phytochemicals affect nutrient digestibility and/or feed intake. For example, feeding animals a diet supplemented with a 100 mg/kg phytochemical blend (containing 18% thymol and cinnamaldehyde) increased their daily weight gain significantly in comparison to the un-supplemented basal diet [68]. A similar positive influence was observed on nutrient digestibility detected after 4 or 5 weeks of continuous feeding [68]. However, feeding the same dose of phytochemicals for a shorter period of time (1 week) did not show any effect on the animals involved in the experiment [79].

Similar to the duration factor above, another study showed the influence of the administered dose on the varying responses of animals. Feeding a diet supplemented with a 2000–3000 mg/kg phytochemical blend (containing 60 mg/kg carvacrol and 55 mg/kg thymol) for 3 consecutive weeks increased feed intake and the reported average daily gain of weaning pigs in comparison to animals that were fed with un-supplemented basal diet [80]. However, a lower level of supplementation (at 1000 mg/kg) did not show any enhancement of performance [80].

In addition to their antimicrobial capabilities, some phytochemicals exert anti-oxidative and anti-inflammation functionalities [64]. A commercial blend of phytochemicals containing oregano essential oils [81] and cinnamaldehyde [82] was shown to increase the expression of nuclear factor-erythroid 2-related factor-2 (Nrf2) and thereby induce the expression of many other favorable enzymes providing an advantageous oxidative-stress defense mechanism within the intestinal epithelia. Moreover, the ability of some phytochemicals to inhibit the nuclear factor κB (NF-κB) can explain their reported anti-inflammatory activities [81, 83]. Generally speaking, the anti-oxidative and anti-inflammation properties of certain phytochemicals add a beneficial synergistic effect to their antimicrobial capabilities which might aid in addressing some pathophysiological factors/environmental stressors important within the animal production environment [84].

The future development and use of integrated genomic, meta-genomic, transcriptomic and proteomic approaches will help researchers to better understand the modes of action of many phytogenic compounds and possibly lead to the development of feasible additives/treatments that can be used as alternatives to antibiotics in animal feeds.

### Synthetic polymers and nanoparticles and their potential use within the animal-production chain

In our battle against antibiotic resistance, polymer and material scientists have an important role to play. These scientists are contributing to the mitigation of antibiotic resistance through the use of major strategies that include: (a) developing antibiotic alternatives using antimicrobial metal-nanoparticles and antimicrobial-polymers which bacteria are less likely to develop resistance to and (b) fabricating new nano-carriers to boost the therapeutic efficacy of existing antimicrobial molecules with an increased selectivity to inhibit bacterial cells rather than host eukaryotic cells.

Scientists have made rigorous attempts to increase zinc bioavailability as well as its influence on growth performance and immune response by using lower doses/concentrations of zinc in feeds and more recently by utilizing ZnO nanoparticles (NPs) [85–87]. While these efforts were met with a certain degree of success, major challenges remain such as fully characterizing these ZnO NPs and providing direct evidence of their nanostructures; such pivotal information is still missing at large from the published body of literature.

Cationic antimicrobials have been circulating within general-use in clinical and domestic settings since 1955. The most well-known cationic antimicrobials are quaternary ammonium compounds (QACs). These cationic surfactants contain one quaternary-nitrogen associated with at least one major hydrophobic substituent. The mode of action of QAC against bacterial cells is hypothesized to involve a general perturbation of the lipid bilayer membranes (including bacterial cytoplasmic membranes and the outer-membranes of Gram-negative bacteria). Following a tight ionic association between the positively charged quaternary-nitrogen in QAC and the negatively charged phosphate groups in membrane phospholipids, the hydrophobic tail of QAC intertwines into the hydrophobic membrane core to decrease membrane fluidity and disturb its osmo-regulatory functions. Since the above mechanism involves QACs, penetration into the core of bacterial membranes to impact their structures, QACs can practically be pumped out by bacterial ATP-dependent efflux pumps (as a defence counter response) and be less efficient in killing bacteria in some cases.

The most recently developed and reported cationic polymers can impact the fluidity of bacterial membranes (hence their physiological functions) by interacting specifically with the phospholipid bilayer without being susceptible to the action of efflux pumps-mediated resistance mechanisms. Poly(hexamethylene biguanide) (PHMB) is a good example of this as it has been used extensively for skin disinfection, wound-infection control, and swimming pool disinfection for several decades, and so far no bacterial resistance to PHMB has been ever reported [88]. To become resistant to cationic polymers such as PHMB, a bacterium would need to completely change the chemistry of its membrane, which is unlikely to happen.

Xue et al. [89] tested the ability of a highly-branched cationic polymer poly(amidoamine) (PAMAM) in inducing bacterial resistance. After 15 successive subcultures were conducted, the MIC values of the tested PAMAM for *S. aureus* (ATCC 29213) and an extended-spectrum beta lactamase-producing *Escherichia coli* (ATCC 35218) were unchanged indicating a lack of induction of any antibiotic resistance genes. In contrast, the MIC values of classical antibiotics (such as ceftazidime, ampicillin, levofloxacin, oxacillin, and erythromycin) were increased by 8- to 64-folds after the 15 successive subcultures were conducted reflecting the emergence of resistant mechanisms due to the bacterial passaging/adaption.

In order to have value as therapeutic agents, in addition to the absence of resistance induction, cationic polymers must selectively target pathogens rather than cells belonging to the host. The selectivity of an antimicrobial agent is generally defined as the ratio of its hemolytic concentration (the concentration that kills 50% of red blood cells) to its MIC against a chosen pathogen. Lienkamp et al. [90] used a molecular “construction kit” to independently combine cationic and hydrophobic moieties into polymers aiming at assembling a series of amphiphilic antimicrobial polymers with refined activities and selectivity. Starting with a series of possible candidates, they successfully screened out an efficient antimicrobial polymer with an enhanced selectivity value (as high as 533) for *S. aureus* over red blood cells.

Based on the results of the above numerous studies and in view of their low potential for inducing bacterial resistance, antimicrobial polymers hold the promise as key alternatives to antibiotics within veterinary medicine/animal production applications in the coming decades.

Nano-carriers can be used to not only boost the therapeutic efficacy of existing antimicrobials [91] but also to convert potent broad-spectrum non-selective biocides into selective ones, therefore allowing their safe use as alternatives to antibiotics. Liu’s group designed and synthesized a potent antimicrobial agent, [3-(4,4-dimethyl-2,5-dioxo-imidazolidin-1-yl)-propyl]-dimethyl-tetradecyl-ammonium chloride (C17), which induced a 6 log reduction of MRSA within 10 min at a concentration of 188 ppm without inducing any resistance in *P*. *aeruginosa* [92]. However, even a 66 ppm dose of C17 can wipe out >95% of human fibroblasts within 10 min of contact. The high cytotoxicity of this efficient biocide, which comes without any issues connected to bacterial resistance-development, limits its actual applications. The encapsulation of C17 into a solid lipid nanoparticle (SLN) followed by a conjugation step of the resulting matrix to a specific anti-MRSA antibody resulted in increasing the selective efficacy of C17 toward MRSA while reducing its cellular toxicity in fibroblasts [93].

In a MRSA and fibroblast co-culturing assay, 2 mg/mL of encapsulated and antibody-conjugated C17 nanoparticles wiped out all MRSA cells in 2 h while preserving 50% of total fibroblast cells in comparison to >98% reduction of both fibroblast and MRSA cells in the case of free C17 at an equivalent concentration. In the above example, a nanoparticle assembly exhibiting selective antibacterial activity towards a specific pathogenic bacterium has been successfully developed starting from a non-selective and potentially non-resistance-inducing biocide, C17. The authors suggested that the binding of C17-SLN-Ab to MRSA cell-surface is most likely facilitating its endocytosis hence the later release of C17 inside MRSA cells, leading to a subsequent effective and selective inactivation of MRSA. Importantly, by switching the conjugated antibody, they were able to turn the same nano-assembly to be more specific towards another target bacterium, *E. coli* K12, demonstrating the versatility of this strategy.

The above concept can be extended to other potent antimicrobial agents, against which bacterial resistance is unlikely to develop. Because the antibody itself does not impact the viability of the bound bacterium and the encapsulated biocide kills the bacteria in a non-specific way; it is intrinsically less likely to induce bacterial resistance or select any resistant mutants. Overall, this study opens a new door of killing bacterial pathogens while potentially bypassing bacterial resistance mechanisms by using the most recent advances in nanotechnology.

In essence, understanding the mechanism(s) of bacterial growth-inhibition of many novel synthetic polymers and nanoparticles should provide a safe, effective, and inexpensive way to control the prevalence of antibiotic resistance in the animal production chain.

## Enzymes

The increasing acceptance of using recombinant proteins/enzymes to enhance animal productivity [94] has led to a serious consideration of their application in addressing the increased use of antibiotics in animal farming. Among the most promising examples are the use of glucose oxidases, alkaline phosphatases and proteases.

### Glucose oxidase

**Figure 2:**
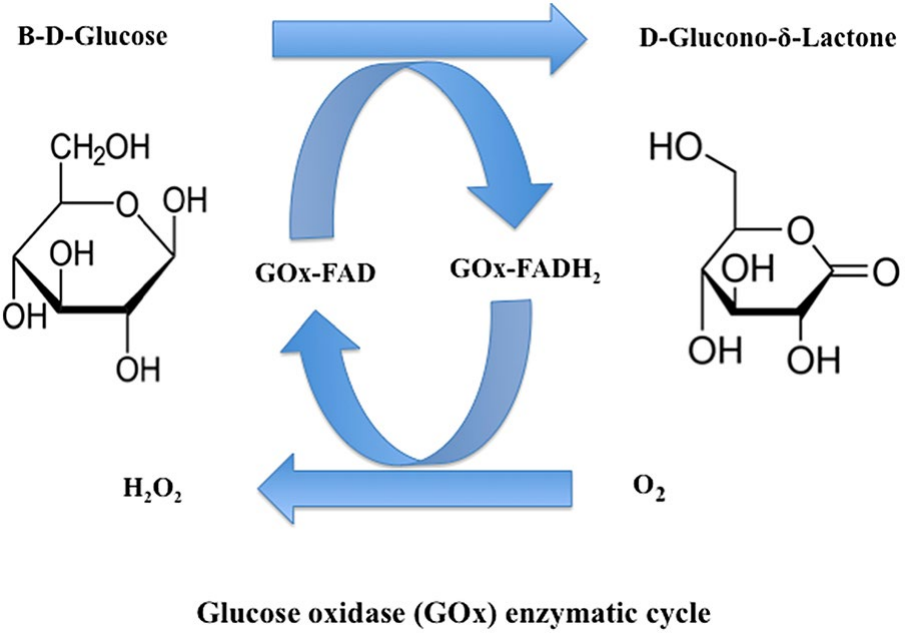
**The enzymatic cycle of glucose oxidases (GOx) and the production of hydrogen peroxide.** In the above system, GOx which is a flavin adenine dinucleotide (FAD)-dependent enzyme oxidises β-d-glucose to gluconolactone through a process that involves the transfer of two protons and two electrons from the substrate to the FAD cofactor. By passing these to oxygen, hydrogen peroxide is formed locally within the surrounding environment.

The antibacterial property of a glucose oxidase is largely dependent on its ability to generate and accumulate hydrogen peroxide (H_2_O_2_) in the surrounding environment by a glucose oxidase-mediated conversion of glucose (Figure 2). This enzymatic activity has been exploited for centuries in addressing bacterial infections, in wounds for example, through the well-established practices of alternative medicine.

Most recently, a patented preparation containing a glucose oxidase (in addition to other active ingredients) was shown to inhibit a number of pathogenic bacterial species and prevent biofilm formation of *S. aureus*, methicillin-resistant *S. aureus*, and *P. aeruginosa*. The obtained results, interestingly, showed that *Staphylococcus* cells were more susceptible to the glucose oxidase treatment than the *P. aeruginosa* cells [95]. The results were encouraging.

Another study looked into developing a H_2_O_2_ generation system using a glucose oxidase and glucose to target *Propionibacterium acnes*. The results indicated the effectiveness of such treatment in reducing the observed inflammation over both short and long-term applications at 68 and 56% rates, respectively [96].

The aforementioned examples show that a recombinant glucose oxidase can be utilized in the inhibition of bacterial biofilm(s) formation and any associated symptoms (such as inflammation), as well as demonstrating the potential of this treatment for generating topical ointments for animal treatment purposes. The ability to apply such intervention through oral routes needs to be further investigated and developed.

### Alkaline phosphatase

The digestive-system microenvironment plays a pivotal role in sustaining animal health and productivity. Many factors including the consumed feed, gut microbiota, and the animal’s intestinal immune response interrelate to influence this localized environment. Among the recently highlighted risk factors that influence the homeostasis of such an environment are bacterial lipopolysaccharides (LPS) that have long been recognized as potent pro-inflammatory mediators/toxins that constitute a challenging-factor for growing animals [97, 98]. Alkaline phosphatases are considered to be potential vehicles for detoxifying LPS within this environment [99]. The endogenous alkaline phosphatase (IAP) enzyme usually localizes to the apical brush border [99] and participates in the de-phosphorylation of bacterial LPS in addition to un-methylated cytosine-guanosine dinucleotides and flagellin leading to a reduced bacterial toxicity and inflammation responses [100].

In animals, the endogenous levels of IAP are reported to decrease at the weaning stage; hence pathogenic gram-negative bacteria (through an LPS-mediated mechanism) can up-regulate inflammatory responses leading to a symptomatic diarrhea [101]. To address this issue, the use of exogenous IAP over-expression systems to modulate the animal’s overall IAP levels, promote gut health, and reduce the associated diarrhea has been suggested.

In a recent study the effect of an intestinal alkaline phosphatase (IAP) and sodium butyrate on LPS-induced intestinal inflammation was evaluated in pigs. The exogenous IAP was able to complement endogenous IAP levels and down-regulate LPS-induced inflammatory responses via the RelA/p65 (NF-κB) route demonstrating that such a treatment may indeed be beneficial in attenuating LPS-induced intestinal inflammation [102].

### Proteases

Increasing evidence points to the use of exogenous protease(s) to reduce the protein content of diets and improve nutritional and health outcomes of farm animals. This practice is hypothesized to lower the indigestible part of feed, to limit subsequent hindguts fermentations and the potential growth of enteric pathogens, and to improve the gastrointestinal tract overall health (higher villi, etc.). Furthermore, it is predicted that the use of proteases can indirectly reduce the usage of antibiotics by improving gut and animal health and reducing stress-related hormones. This idea is supported indirectly by a recent study that investigated the effects of dietary protein levels on disease-resistance and immune function of grass-carp (*Ctenopharyngodon idella*) after challenging with *A. hydrophila* [103]. The inclusion of proteases led to optimized levels of dietary proteins, enhanced disease-resistance, and immune function while up-regulating antimicrobial peptides and anti-inflammatory cytokines. At the same time it down-regulated pro-inflammatory cytokine mRNA levels. Similar results were observed after the grass-carp was challenged with *Flavobacterium columnare* in another study [104].

A recent study was conducted to investigate the effect of dietary proteases on nutrient digestibility, growth performance, crude protein digestibility, enzyme (pepsin, pancreatic amylase and trypsin) activities, plasma total proteins, intestinal villus heights, intestinal morphology and the expression levels of specific genes. Significant increases in growth performance have been observed which were attributed to better intestinal development, enhanced protein digestibility, and improved nutrient transport efficiencies [105]. The supplementation of proteases (200 and 300 mg/kg) within the diet increased the ratio of villus heights to crypt depth significantly, especially in the duodenum, jejunum and ileum, and induced higher expression levels of the peptide transporter 1 (PepT1) within the duodenum region [105].

The influence of a commercial acid-stable protease mixture on the utilization of corn and soybean meal-based diets was evaluated most recently. The grouped animals had free access to a mash diet and the ileal content was collected for digestibility determination after 14 or 29 days of the protease-mixture administration. The results collectively showed that the apparent ileal digestibility of individual amino acids was increased during the supplementation course (29 days of treatment) while the apparent ileal digestibility of the indispensable amino acids, methionine and cysteine, and branched-chain amino acids was increased only by the end of the study period but not in an earlier phase. The presented data argues for a continuous supplementation regimen of proteases during the entire production phase to maximize the associated benefits and overall animal productivity [106].

A further exploration of the effects associated with supplementing broilers diets with exogenous proteases to enhance nutrient digestibility in low-protein poultry by-product meal based diets was conducted. The continuous dietary supplementation of these poultry by-product meal diets with exogenous proteases for 35 days increased feed intake and body weight gain in treatment animals. The overall improvements in feed intake and body weight gain were associated with the provided levels of crude proteins during that period which also influenced nitrogen retention rates. The presented results suggested that the addition of enzymes not only improves production parameters, but crude protein levels can also be safely reduced while using poultry by-product meals supplemented with exogenous proteases for the same production outcomes [107].

The use of protease supplementation for enhancing the nutritional and production outcomes of a diet reduced in crude protein as well as digestible amino acids and energy was recently reported. The suggested protease supplementation (at 125 g/ton) showed the potential to maintain the live weight and feed conversion rate of pigs up to the level of a matching regular diet [108]. Furthermore, the use of exogenous proteases and their effects on the digestion of dietary proteins was evaluated in another study [109]. A commercial protease enzyme (200 g/ton) was used to enhance corn-soybean based diets and track animals’ performance for 42 days. The growth performance in terms of body weight and average daily gains were increased significantly while gains per feed remained the same. Moreover, the apparent total tract digestibility and NH_3_ emissions were enhanced. Blood samples profiling indicated decreased creatinine levels without any changes in the lymphocytes or red/white blood cells counts. Fecal microbiota (particularly *E. coli* and *Lactobacillus*) were also similar between all groups. From the aforementioned studies [108, 109], the influence of protease supplementation on noxious gas emissions and creatinine seems also to be more age- or phase-related.

### Non-starch polysaccharides hydrolytic enzymes

The use of non-starch polysaccharide (NSP) hydrolytic enzymes in boosting antibiotic treatments within the digestive systems of mono-gastric animals that cannot ferment and utilize such polysaccharides is another promising approach. This technique is similar, in principle, to the use of proteases. In essence, diets with high concentrations of rye and barley have high viscosity when passing the alimentary canal due to their high content of non-starch polysaccharides (NSP)/fibers [110]. The elevated viscosity traps many present pathogens and hinders any administered antibiotics from reaching their targets, hence increasing the proliferation of pathogenic bacteria. When enzymes capable of breaking down these non-starch polysaccharides are used, the matrix viscosity is lowered letting the used antibiotics reach the involved bacterial species to exert their bactericidal effects [111].

An earlier study established the effects of an exogenous supplementation of non-starch polysaccharides hydrolytic enzymes on the activities of endogenous pancreatic and small intestinal digestive enzymes in animals fed with high percentages of barley. Such supplementation (at the 0.15% w/w level) improved the growth performance of the studied animals and had no effect on the digestive-enzyme activities within the pancreas but at the same time the treatment affected the duodenal contents where the proteolytic enzymes, trypsin, amylase, and lipase activities decreased by 57.56, 76.08, 69.03 and 40.22%, respectively [112].

### Yeast cell walls and mannan oligosaccharides

In the past decade, mannan-oligosaccharides have been reported as alternatives to antibiotics with benefits that include an improvement in the animal’s performance, an enhancement in the feed conversion efficiency, and a boost of the gastrointestinal health. Despite these benefits, the molecular mechanisms behind such functionalities are not yet clear.

To elucidate the effects of these additives on the intestinal gene expression and characterize the involved biological pathways, a recent study was conducted in which young broilers were fed a diet supplemented with 2.2 g/kg of mannan-oligosaccharides for 3 consecutive weeks followed by jejunal gene-expression profiling [113]. The obtained results showed the ability of the involved supplement(s) to influence more than 670 genes clustered in pathways related to energy production, cell death, and protein translation. Genes related to the cellular oxidative-phosphorylation in particular were among the most upregulated targets in addition to other genes connected to the cellular stress response such as peroxiredoxin 1, superoxide dismutase 1, and thioredoxin [113].

Another study reported the beneficial effect of mannan-oligosaccharides on inhibiting the adherence of bacterial cells including *C. jejuni* and *C. coli* [114]. Other profound effects in correlation to intestinal morphology in particular were reported in which the inclusion of β-galactomannans in chicken diets reversed the negative effects of *Salmonella* Enteritidis infections [115].

Among the good (and cheap) sources of mannan-oligosaccharides is dried brewer’s yeast with contents close to 5.2%. The dried brewer’s yeast was assessed most recently as an alternative to the antimicrobial carbadox for young pigs. Agglutination tests confirmed the ability of the yeast product to sequester/bind several serovars of *E. coli* and *S. enterica* [116]. Pigs that were fed with 3% yeast content showed reduced total fecal coliforms throughout the entire post-inoculation period with a reduced colonization of total coliforms in the duodenum, jejunum, cecum, and colon. Moreover, serum immunological traits were enhanced in the pigs that were fed the yeast blend [116].

The results of these studies collectively suggest that mannan-oligosaccharides could be used as a feed supplement for livestock to control enteric infections, to improve performance, and to minimize the use of antibiotics as growth-promoters throughout different cellular mechanisms.

## Challenges and future prospects for innovative drugs, chemicals, and enzymes in animal production

The development of novel agents for pathogenicity control, especially anti-virulence ones, requires an in-depth understanding of the unique roles that such virulence factors have in disease/infection progression. Without such knowledge, the possibility of off-targets cannot be ruled out. The lengthy process of conceptualizing a detailed mechanistic understanding of virulence factors and the research resources needed for such conceptualization are sometimes considered to be major limiting factors.

Another challenge related to the use of recombinant enzymes (e.g. glucose oxidases) is the fact that such enzymes exhibit considerable variations in their antibacterial activity/efficiency based on the surrounding environment. Any factor that influences the overall H_2_O_2_ content, accumulation, or diffusibility within the matrix will retrospectively affect the treatment/usage outcomes.

In a recent study, the antibacterial activity of honey samples (a rich source of glucose oxidases) against *P. aeruginosa* isolates significantly correlated with H_2_O_2_ content and more importantly, with the glucose oxidases levels. Higher glucose oxidases levels led to better outcomes. In response to this finding, the authors proposed the breeding of novel honeybee lines that express higher levels of glucose oxidases in order to increase the antibacterial efficacy of the final products [117].

While the use of proteases is surely a promising way of inhibiting bacterial biofilm(s) formation and accelerating infection clearance, the use of proteases is by itself a controversial topic. Some recent reports indicated unwanted roles of bacterial proteases in extending bacterial infections within chronic wounds environments [118]. The elevated proteolytic environment within such wounds, initiated by bacterial metallo-proteinases, is believed to be one among many legitimate reasons standing behind the failure to heal. Moreover, recent findings indicated that bacterial proteases actively participate in establishing the colonization process and further facilitate the evasion of immune systems [118]. Altogether, the disruption of bacterial biofilm(s) might appear to a tangible strategy for addressing bacterial pathogenicity, especially if used in combination with an effective antibiotics treatment. However the possibility of lower antibiotics doses/concentrations to achieve more efficient outcomes is deemed risky by many experts as the disruption of bacterial biofilm(s) leads, in certain cases, to the dissemination of bacterial cells and colonization of newer sites [118].

The use of polymers and surface-coating technologies for quorum-sensing manipulation is considered to be very potent technology but attention should be paid to the conjugation of the involved compounds in a way that retains their ability to influence bacterial hosts/colonies [119]. Investigating the key chemical features of quorum-disruptors in addition to best available linkers is possibly the way to successfully obtain modified surfaces with desirable bactericidal functionalities [119].

While the rapid evolution and spread of antibiotic resistance mechanisms among animal pathogens is outpacing the discovery of new antibiotics, there is an increasing number of published studies clearly showing that alternatives do exist. The optimization of many of the approaches outlined in this review coupled with a deep understanding of their underlying molecular mechanisms will eventually lead to strong and empirical strategies for mitigating antibiotics usage in the field of animal production and husbandry.

## Conclusions

In the past 2 decades, many promising approaches have been reported for addressing bacterial resistance and antibiotics over-usage in the fields of animal farming and livestock production (Table 1). While these approaches have the potential to solve such long-lasting dilemmas, it seems that no single approach will be able to completely replace antibiotics use in farming. In reality, it is more likely that a combination of approaches will achieve the needed breakthroughs in this area. The increasing public interest in replacing antibiotics (or at least decreasing their usage) will ultimately lead to a reduced dependence on these agents and the diminished possibilities of a superbug emergence within the foreseeable future.

**Table 1:** **Some of the most explored antibiotic alternatives with the potential of feasible usages within the animal production chain**

Alternative	Mode of action	Advantages	Challenges	Continuous use
Drugs targeting quorum-sensing	The disruption of bacterial quorum-sensing	Specific targets	Expensive	No, as concerns for developing resistance do exist
Recombinant enzymes	Varying mechanisms that span the generation of H_2_O_2_ to the enhancement of dietary protein digestibility	Varying targets with the possibility of combining more than one mechanism in parallel	Expensive	Yes, without any concerns of resistance-development
Phytochemicals/botanicals	Varying mechanisms	Low costs	Varying outcomes and efficiencies	Yes, without any concerns of resistance development
Minerals	Production of hydroxyl radicals and/or reactive oxygen species	Most effective	High concentrations are needed	No as concerns for environmental contaminations with heavy metals exist
Organic acids/acidifiers	The un-dissociated acids diffuse across cell-membranes destroying the cytoplasm of pathogens or inhibiting their growth	Low costs	Varying outcomes and efficiencies based on the used compound	Yes, without any concerns of resistance-development
Yeast cell walls and mannan-oligosaccharides	Inhibit the adherence of bacterial cells	Low costs	Not fully validated/tested	Yes, without any concerns of resistance-development
